# Plant Availability of Magnesium in Typical Tea Plantation Soils

**DOI:** 10.3389/fpls.2021.641501

**Published:** 2021-08-10

**Authors:** Qunfeng Zhang, Dandan Tang, Xiangde Yang, Saipan Geng, Ying He, Yupei Chen, Xiaoyun Yi, Kang Ni, Meiya Liu, Jianyun Ruan

**Affiliations:** Tea Research Institute, Chinese Academy of Agricultural Sciences, Hangzhou, China

**Keywords:** Mg fertilization, tea plant, Mg efficiency, plant availability of Mg, tea plantation soils

## Abstract

**Background and Aims:** Magnesium (Mg) fertilizer has been proved to play an important role in improving the yield and quality of tea. However, plant availability of Mg, including its use, efficiency, and quality improvement effects, were highly affected by plant species, soil characteristics (nutritional status, etc.), and Mg status (chemical-available, etc.).

**Methods:** Tea plants were pot-cultivated in 12 typical tea plantation soils amended with and without Mg fertilizer. Exchangeable Mg (Ex-Mg) concentration in soils was quantitatively extracted using four extraction solutions (Mehlich-3, BaCl_2_, CaCl_2_, and NH_4_OAC). Plant availability of Mg was evaluated by Mg uptake and its use efficiency, as well as its association with quality components in tea plants.

**Results:** Ex-Mg in soils was extracted most efficiently by Mehlich-3, while Mg concentrations in tea plant tissue were higher correlated with Ex-Mg extracted by CaCl_2_ than other extraction solutions. Mg fertilizer use efficiency in tea plant varied from 6.08 to 29.56 %, and the effect of Mg application on tea quality improvement and the use efficiency of Mg fertilizer both negatively correlated with total Mg concentration (*r* = −0.94 and −0.63, respectively) and nitrogen (N) level (*r* = −0.61 and −0.51, respectively) in soils prior to tea plant cultivation.

**Conclusions:** CaCl_2_ could be recommended for plant-available Mg extraction in tea plantation soil, and Mg fertilizer use efficiency could be affected and predicted by total N and Mg status in soils prior to tea plant cultivation, providing a potential theoretical for the guidance of Mg fertilization for tea yield and quality improvement in tea plantation management.

## Introduction

Magnesium (Mg) as the second most abundant intracellular cation has been proven to have vital functions in plants because Mg participates in chlorophyll synthesis and catalytic action (Gerendás and Führs, 2013). In recent years, considerable progress has been made in studying the effect of Mg fertilization on the yield and quality of cash crops (Cakmak, 2013; Dechen et al., 2015). In tea plants, a previous study has shown that the use of Mg fertilizer can effectively improve the yield and quality of tea (Ruan et al., 1999). Our early experiment found that Mg application significantly increased caffeine content and aroma components in tea products (Ruan et al., 1999). Besides, Ruan et al. (2012) also suggested that improving Mg nutrition in tea planting effectively decreases the ratio of total polyphenols to amino acid (TP/AA), which is positively and strongly connected to green tea taste (Zhang and Ruan, 2016). However, there is a deep gap in the knowledge of the requirements of crops for Mg (Grzebisz et al., 2010).

Soil Mg incorporated in the crystal lattice structure of minerals cannot be directly absorbed by plants (Senbayram et al., 2016). In general, plants are prone to absorb the Mg readily from the soil solution. Exchangeable Mg is an important source of plant-available Mg. In the agricultural production system, the availability of Mg to crops depended on various factors such as soil texture, cation exchangeable capacity (Hariadi and Shabala, 2004), site-specific climatic and anthropogenic factors, agronomic management practices, and the crop species itself (Mikkelsen, 2010).

Adequate soil Mg is the key to ensure robust crop growth and production, and soils with low concentrations of Mg can decrease agricultural productivity and quality (Wang et al., 2020). Mg losses by mobilization and leaching in the soil (Schachtschabel, 1954) or Mg depletion due to intensive crop production (Pol and Traore, 1993) causes Mg deficiency in soil. Additionally, cationic competition, resulting from long-term imbalanced soil fertilization, causes nutrient heterogeneity in soils. A good soil Mg condition is the prerequisite to ensure Mg uptake by crop roots and enhance Mg utilization efficiency. Mg deficiencies in soil are a major limiting factor for crop growth (Cakmak and Yazici, 2010; Cakmak, 2013; Guo et al., 2016), particularly in acidic soils that are highly saturated with cations, such as H^+^, Al^3+^, and Mn^2+^, and which suffer intensive leaching, particularly in areas with high rainfall totals (Mayland and Wilkinson, 1989; Wilkinson et al., 1990; Gransee and Führs, 2013). Tea plantations are generally located in regions with acidic soil, and Mg deficiency is an important limiting factor for tea production, both in terms of yield and quality (Wu and Ruan, 1994). At present, however, the input of Mg fertilizer to tea plantations is insufficient (Ruan et al., 2002; Ni et al., 2019).

The availability of Mg also depends on the activity or proportion of Mg relative to soluble and exchangeable amounts of K, Ca, Na, Al, and Mn. Previous studies have reported that the status of nutrients such as potassium (K) (Farhat et al., 2013) or nitrogen (N) (Ruan and Gerendas, 2015) is closely related to the biological effects of Mg application (Lasa et al., 2000). The cooperative of Mg and N nutrition on crops has been widely reported (Ruan et al., 2000, 2004). For example, a hydroponics experiment with tea plants showed that the effects of Mg on tea quality were limited by N nutrition (Ruan et al., 2012). Moreover, Mg supply could improve the absorption and metabolism of nitrate in tea plants (Ruan et al., 1998) and promotes the long-distance transportation of amino acids and sugars in the xylem and phloem of tea plants (Ruan et al., 2012). Excessive application of high rates of K^+^ and ${\text{NH}}_{4}^{+}$ fertilizers, antagonistically interferes with plant Mg uptake, thus enhancing the risk of Mg deficiency.

In recent years, studies of the molecular mechanisms of Mg metabolism (Deng et al., 2006) and the need for Mg in the field have produced much new knowledge (Conn et al., 2011; Senbayram et al., 2016). In particular, the understanding of the plant availability of Mg for plant growth has deepened (Gransee and Führs, 2013). However, the challenge is to relate the data obtained from soil analysis to phyto-availability and plant growth (Kopittke and Menzies, 2007). Nutrient balance is considered as a simple diagnostic procedure, evaluating the current status of crop nutrient management. Two main approaches, the soil surface balance (SSuB), and the soil system balance (SSyB), are used to assess trends in a nutrient balance during a fixed period. SSuB relies on a net balance of an external input and output of a given nutrient, while SSyB relies on both external and internal (soil) resources (Oenema et al., 2003). In addition, the efficiency of a given extraction is shown by the correlation between the extractable Mg content and crop uptake. A good extraction should simulate the capacity of the roots to uptake nutrients, extract as many elements of interest as possible, and be suitable for the prediction of extractable Mg in chemically and physically different soils (Van, 1988). The Mehlich 1 acid solution (Nelson et al., 1953), Mehlich-3 solution (Mehlich, 1984), and ion-exchange resin (IER) (Van Raij et al., 1986) are commonly used as extractants. In some cases, the Mehlich-3 solution has shown a better correlation between extracted P and plant uptake when compared with Mehlich 1 (Tran et al., 1990).

In this study, to evaluate plant availability of Mg under different soil nutrients status, we collected soils from 12 tea-producing areas of China to cultivate tea plants in a greenhouse supplied with and without the additional Mg nutrient. Our main hypothesis is that the plant-available Mg in tea plantation soil depends on the Mg status (chemical-available, etc.) and soil characteristics (nutritional status, etc.).

## Materials and Methods

### Experimental Design

Surface soils (0–20 cm) were collected from 12 typical tea plantations located in main tea producing areas in China; the detailed information of collected soils is shown in Table 1. Prior to the experiment, soil samples were air-dried, ground, sieved, and mixed with quartz (1:1, v:v). Then, four 1-year-old tea clones (Longjing-43) were selected to cultivate in the mixed soils with a 350 ml plastic cup (bottom drainage). All pots were evenly placed in the climate chamber (Supplementary Figure 1). The light intensity was 200 mmol m^−2^ s^−1^ and the photo/dark period was 14/10 h. The air temperature and relative humidity were maintained at 26/22°C in the photo/dark period and 70%, respectively. During the experiment, 50 ml deionized water was added to plots every 3 days and soil leachate was also periodically collected with a plastic cup for recycling use. After 2 months of pre-incubation, tea plants were divided equally into two groups. Experimental groups (+Mg group) supply Mg equivalent to 40 mg MgSO_4_. After 6 months of cultivation, the root, stem, and leaves were sampled, respectively. Plant samples were frozen immediately with liquid nitrogen and freeze-dried, respectively. Root and stem were oven-dried at 60°C and then ground using a ball mill (M301, Retsch, Germany). Additionally, visible roots were removed the remaining soils were then air-dried (Supplementary Figure 2) and passed through a 2-mm mesh sieve to remove the quartz from the soil.

**Table 1:** Soil properties of tea plantations at the selected 12 countries in China.

**Site**	**pH**	***C* (mg/g)**	***N* (mg/g)**	^*^Soil particle-size distribution (%)			**Soil texture**
				**Clay**	**Silt**	**Total sand**	
ChengDu (CD)	3.78	112.77	11.12	15.54	65.88	18.58	Silty loam
GuiLing (GL)	4.25	121.64	10.41	57.73	24.20	18.06	Clay
HuangGang (HG)	3.58	215.03	22.20	14.25	59.54	26.22	Silty loam
XinYang (XY)	4.73	120.04	11.63	27.58	55.71	16.32	Silty Clay loam
XiShuangBanNa (XSBN)	4.93	224.05	16.40	1.63	56.94	40.81	Silty loam
WuXi (WX)	3.84	126.56	10.90	53.95	20.74	25.30	Clay
YiChang (YC)	4.64	158.27	14.52	16.23	61.70	22.06	Silty loam
ChangSha (CS)	4.04	136.02	14.01	0.81	38.14	60.46	Sandy loam
NingDe (ND)	3.76	122.60	12.70	48.27	42.03	9.37	Clay
QingYuan (QY)	4.78	151.70	16.14	39.58	48.03	12.39	Silty clay
XiangXi (XX)	4.47	257.43	24.50	45.82	39.45	14.73	Clay
ChongQing (CQ)	3.84	114.65	11.52	42.76	41.69	15.54	Silty clay

* *Clay(<2 μm), Silt (2–50 μm), Total sand (50–2000 μm)*.

### Analysis of Soil Samples

#### Soil pH, Particle Size Distributions, Total Carbon (C), and N Contents

Soil pH was measured at a ratio of 1:1 (w/v) in deionized water with an ORION 3 STAR pH meter (Thermo Fisher Scientific, Waltham, MA, USA). The particle size distributions were analyzed using a laser particle size analyzer (Mastersizer 3,000, Malvern, UK). Total C and N contents were measured with an elemental analyzer. For the dry-combustion procedure, a Vario MACRO (Elementar Analysensysteme, Germany) resistance furnace CNS elemental analyzer was used. Combustion was carried out in a pure oxygen source and the carrier gas was helium. Two hundred milligrams of soil was weighted on an inert thin boat. The typical sample combustion train started with combustion at 1,150°C in a pure oxygen stream. Each soil had three replicates, and three technical replicates were calculated for mean values.

#### Total Mg and Exchangeable Mg

To measure total Mg content in soils, 0.2 g soil (passed through a 100-mesh sieve) was digested with 5 ml nitric acid, 1 ml perchloric acid, and 2 ml hydrofluoric acid at 140°C for 3 h. Mg concentration in the solution after dilution was analyzed using an inductively coupled plasma atomic emission spectrometry (ICP-AES, Thermo Jarrel Ash, USA).

The exchangeable Mg (Ex-Mg) content was extracted with 0.01 *M* BaCl_2_, Mehlich-3 solution, 1 *M* CaCl_2_, and 1 *M* ammonium acetate, respectively, at a ratio of 1:10 (w/v) for shaking 30 min at 180 r min^−1^, and passed through 0.45-μm-sized cellulose–acetate paper filters for the measurement of Mg concentration by ICP-AES (Yang et al., 2018).

### Analysis of Tea Plant Samples

#### Mg Concentrations in Tea Plant

The samples of the tea plant (three biological replicates, each 0.1 g) were digested by 5 ml mixed concentrated acids HNO_3_-HClO_4_ (Ruan et al., 2012) at 140°C in an electric oven. The digestion was then diluted to 25 ml with distilled water, and the nutrients were measured using the ICP-AES.

#### Total Polyphenols and Free Amino Acids in Tea Leaf

To measure total polyphenols, 60 mg finely ground tea plant powder (three biological replicates) was extracted with 3 ml deionized H_2_O in a boiling water bath for 5 min (with vortex mixing for 2.5 min). A Folin–Ciocalteu colorimetric assay, with gallic acid as the reference standard, was used to spectrophotometrically determine its concentration in the filtrate (ISO14502-1).

The free amino acids in tea leaves were measured *via* high-performance liquid chromatography with a diode array detector (HPLC-DAD, Waters, 2,695–2,998), as previously reported by Zhang et al. (2018).

### Data Analysis

Statistically significant differences in mean values were tested using the one-way ANOVA. Tukey's *post-hoc* test was applied for comparison of multiple groups. Pearson correlation and linear regression analysis were tested with SigmaPlot 12.0 (Systat Software, USA). Mg fertilizer use efficiency was concluded with the formula as:

$$UE=\frac{\left(C1\;*\;M1-\;C2\;*\;M2\;\right)+\left(C3\;*\;M3\;-\;C4\;*\;M4\;\right)+\left(C5\;*\;M5-\;C6\;*\;M6\;\right)}{TM}\;*\;100\%$$

where UE denotes Mg fertilizer use efficiency. C1, C3, and C5 denote Mg concentrations of tea root, stem, and leaf, respectively in +Mg group plants. M1, M3, and M5 denote biomass of tea root, stem, and leaf, respectively in +Mg group plants. C2, C4, and C6 denote Mg concentration of tea root, stem, and leaf, respectively in CK group plants. M2, M4, and M6 mean biomass of tea root, stem, and leaf, respectively in CK group plants. TM denotes the total amount of Mg fertilizer applied in each pot.

## Results

### Soil Ex-Mg Concentration Prior to the Experiment

**Figure 1:**
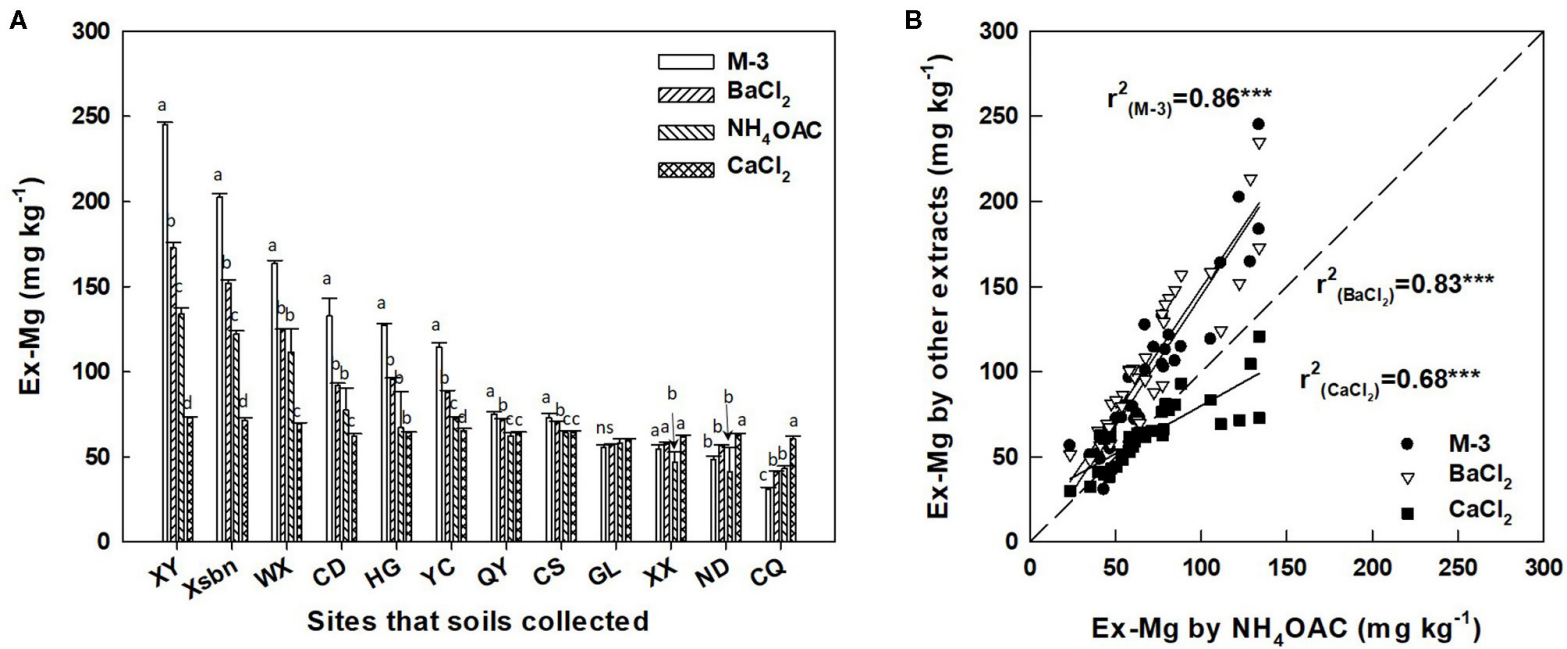
Contents of exchangeable Mg extracted by 1.0 mol L^−1^ CaCl_2_, 0.01 mol L^−1^ BaCl_2_, Mehlich-3, and 1.0 mol L^−1^ NH_4_.OAc in soils pretea cultivation **(A)**, and the correlation of exchangeable magnesium extracted by different extracts **(B)**. Different letters above the bar indicate significant (*p* < 0.01) differences among different extraction. M-3, Mehlich-3 solution; BaCl_2_, barium chloride; NH_4_OAC, ammonium acetate; CaCl_2_, calcium chloride; Ex-Mg, exchangeable magnesium; XY, Xsbn, WX, CD, HG, YC, QY, CS, GL, XX, ND, and CQ indicated that the soil has been collected in the site of Xinyang, Xishuangbanna, Wuxi, Chengdu, Huanggang, Qingyuan, Changsha, Guiling, Xiangxi, Ningde and Chongqing, respectively.

Exchangeable Mg concentration in soils extracted by different extraction solutions varies widely. For example, Ex-Mg concentration extracted by Mehlich-3 solution in XY site was three times more than CaCl_2_-extracted concentration of Ex-Mg (245.04 and 72.85 mg kg^−1^, respectively). However, the concentration of soil Ex-Mg in XY, Xsbn, WX, CD, HG, YC, QY, and CS sits were highly extracted by Mehlich-3 solution and followed by decreasing order of BaCl_2_, NH_4_OAC, and CaCl_2_, while CaCl_2_ solution showed higher soil Ex-Mg extraction efficiency in XX, ND, and CQ sites (Figure 1A). Herein, simple linear regression was applied to model the relationship between the methods of Mehlich-3, BaCl_2_, CaCl_2_, and NH_4_OAC. The results showed that Mehlich-3 solution had the strongest correlation with NH_4_OAC (*r*^2^ = 0.86, *p* < 0.001), followed by BaCl_2_ solution (*r*^2^ = 0.83, *p* < 0.001). In contrast, soil Ex-Mg concentration extracted by CaCl_2_ solution showed a lower correlation with NH_4_OAC (*r*^2^ = 0.68, *p* < 0.001) (Figure 1B).

### Mg Concentration in Tea Plants

**Figure 2:**
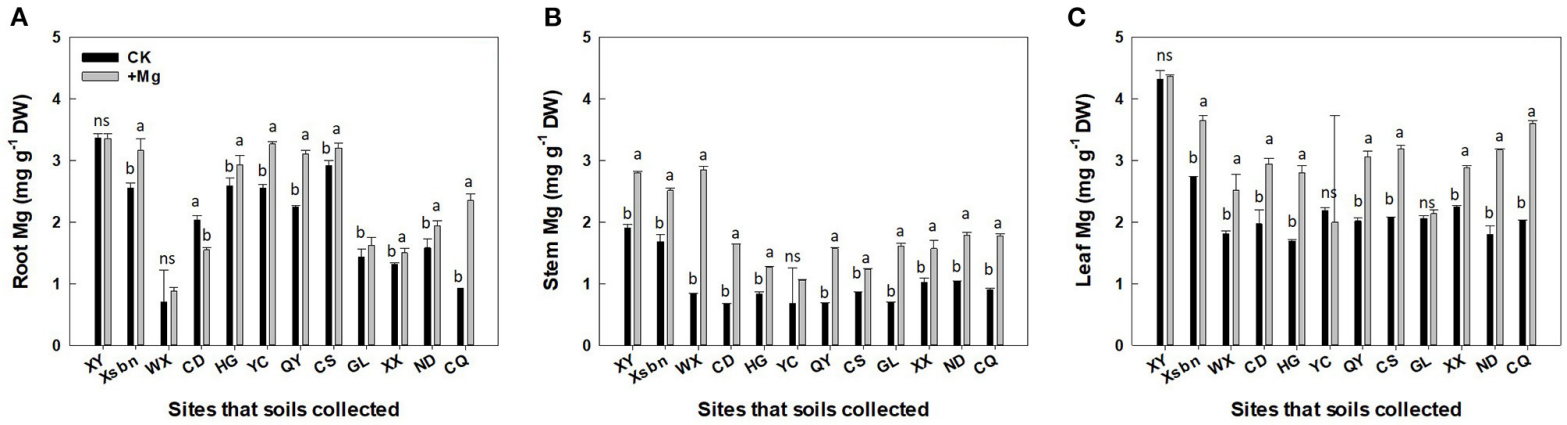
Concentration of magnesium (means ± SD, *n* = 3) in root **(A)**, stem **(B)**, and leaf **(C)** of tea plants. Here “ns” and different letters above the bar indicate insignificant and significant (*p* < 0.01) differences, respectively, between two magnesium treatments. XY, Xsbn, WX, CD, HG, YC, QY, CS, GL, XX, ND, and CQ indicated that the soil has been collected in the site of Xinyang, Xishuangbanna, Wuxi, Chengdu, Huanggang, Qingyuan, Changsha, Guiling, Xiangxi, Ningde and Chongqing, respectively.

Among three tissues, the highest Mg concentration occurred in the leaf, followed by the root and stem (Figure 2). Mg application significantly increased Mg concentration in the roots by 20%, stems (83%), and leaves (36%), respectively (Figures 2A–C). Besides, the effect of Mg application on tea plant varied dramatically due to the initial status of soil Mg nutrition. For example, tea plants cultivated in soils originating from XY site had the highest Mg concentration (3.5, 1.9, 4.3 mg g^−1^ in the roots, stems, and leaves, respectively, Figure 2), which is consistent with Ex-Mg changes in soils (245.6 ± 1.6 mg kg^−1^). In contrast, lower levels of Mg were measured in tea plants tissue (0.92, 0.90, 2.02 mg g^−1^ in the roots, stems, and leaves, respectively, Figure 2) That are cultivated in soils of CQ with less Ex-Mg (30.7 ± 1.5 mg kg^−1^) than XY. Pearson correlation results (Table 2) showed that the Mg concentration in root, stem, and leaf significantly and positively correlated with initial soil Ex-Mg concentration (prior to tea plant cultivation). Compared with root tissue (*r* < 0.6), the Mg concentration in stem and leaf highly correlated with soil Ex-Mg (*r* > 0.6).

**Table 2:** Pearson correlation coefficients between soil exchangeable Mg and Mg concentrations in tea plants (*n* = 36).

**Soil**	**Extract**	**Root Mg**	**Stem Mg**	**Leaf Mg**
Initial	Mehlich-3	0.535^**^	0.638^***^	0.680^***^
	NH4OAC	0.406^*^	0.607^***^	0.640^***^
	CaCl_2_	0.536^**^	0.689^***^	0.661^***^
	BaCl_2_	0.526^**^	0.684^***^	0.698^***^
**Post cultivation**	Mehlich-3	0.499^**^	0.669^***^	0.860^***^
CK group	NH4OAC	0.587^**^	0.707^***^	0.866^***^
	CaCl_2_	0.636^***^	0.733^***^	0.906^***^
	BaCl_2_	0.625^***^	0.679^***^	0.881^***^
Mg group	Mehlich-3	0.447^**^	0.621^***^	0.762^***^
	NH4OAC	0.562^***^	0.547^***^	0.632^***^
	CaCl_2_	0.650^***^	0.640^***^	0.740^***^
	BaCl_2_	0.604^***^	0.613^***^	0.728^***^
ΔMg change	Mehlich-3	−0.428^*^	−0.475^**^	−0.413^*^
(Post cultivation CK Group—Initial)	NH4OAC	0.132	−0.0326	0.108
	CaCl_2_	0.628^***^	0.702^***^	0.917^***^
	BaCl_2_	0.265	0.0666	0.401^*^

* *p < 0.05;*

** *p < 0.01;*

*** *p < 0.001*.

After tea plant cultivation, the nutrient Mg, especially Ex-Mg, could be absorbed directly by the root, and then transported to other tissues like stem and leaf. Indeed, there is a significant correlation (*r* = 0.447–0.906, *p* < 0.01) between Mg concentration among tissue (root, stem, and leaf) and soil Ex-Mg concentration, whether it is in CK group or Mg group (Table 2). Both in the CK group as well as in the Mg group, there was a larger correlation between the Mg concentration in tissue and soil Ex-Mg (*r* = 0.499–0.906). Moreover, soil Ex-Mg concentration extracted by CaCl_2_ had the strongest correlation with Mg concentration in plant tissue (root: *r* = 0.636 and 0.650; stem: *r* = 0.733 and *r* = 0.640; r = 0.906, and *r* = 0.740, in CK and Mg group, respectively).

The changes in soil Ex-Mg pre- and post-tea plant cultivation were defined as ΔMg. The results showed that the Mg concentration in tissue negatively correlated with ΔMg–Mehlich-3 (*r* = −0.428, *r* = −0.475 and *r* = −0.413 in root, stem, and leaf, respectively), but positively correlated withΔMg–CaCl_2_ (*r* = 0.628, *r* = 0.702, and *r* = 0.917 in root, stem, and leaf, respectively). However, there is no significant correlation between Mg concentration in plant tissue and soil Ex-Mg–NH_4_OAC and Ex-Mg–BaCl_2_.

### Use Efficiency of Mg Fertilizer in Tea Plantation Soil

**Figure 3:**
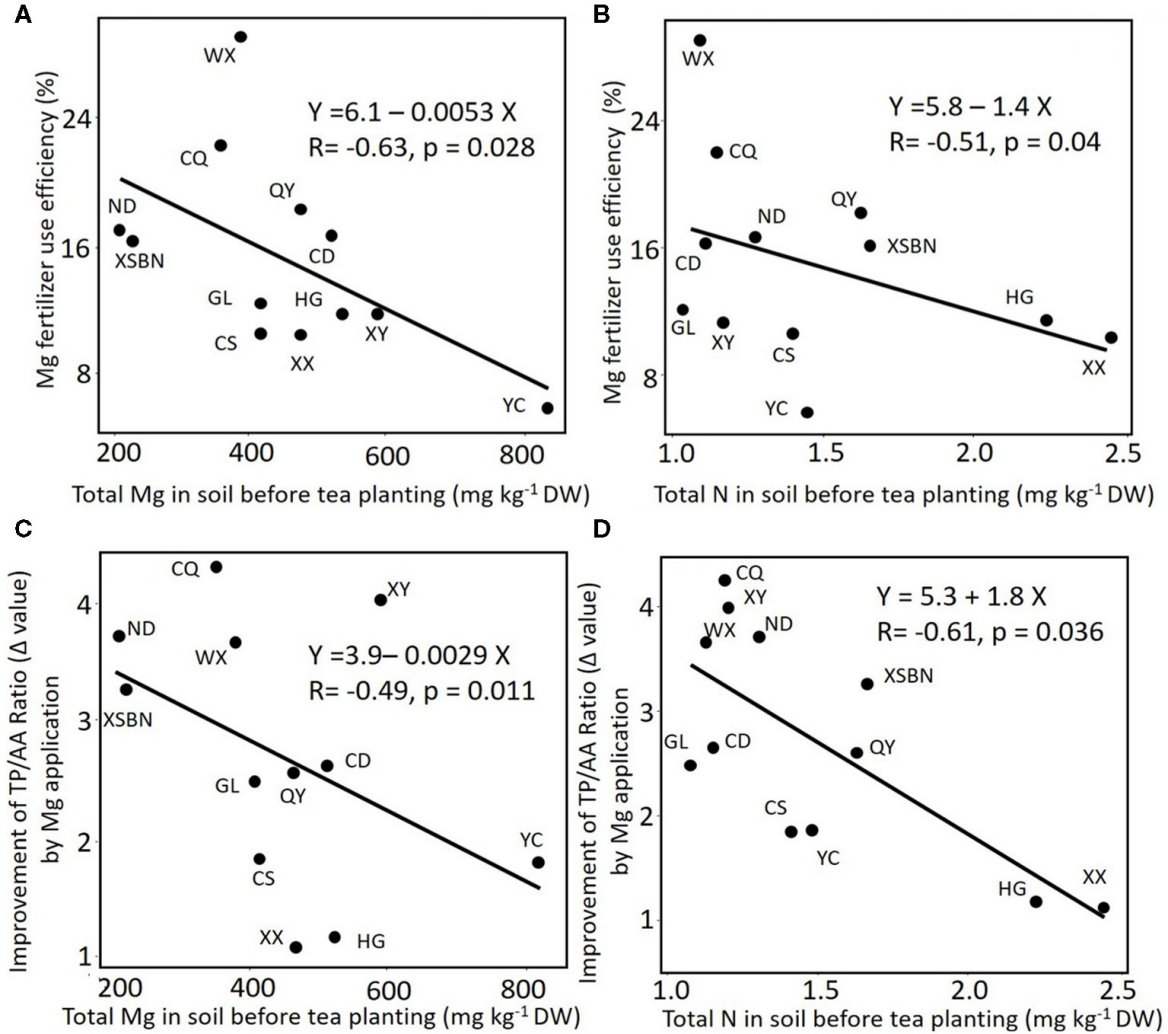
The correlation between content of total magnesium **(A,C)** or nitrogen **(B,D)** in soil and magnesium fertilizer use efficiency **(A,B)** or change of TP/AA ratio (Δvalue) pre- and posttea plant cultivation in tea leaves **(C,D)**. Total Mg was extracted with nitric acid, perchloric acid and hydrofluoric acid from soil. XY, Xsbn, WX, CD, HG, YC, QY, CS, GL, XX, ND, and CQ indicated that the soil has been collected in the site of Xinyang, Xishuangbanna, Wuxi, Chengdu, Huanggang, Qingyuan, Changsha, Guiling, Xiangxi, Ningde, and Chongqing, respectively.

The proportion of Mg taken upped in plant tissue from the Mg supply was 1.30, 10.17, and 3.84%, in root, stem, and leaf, respectively (Table 3). Among 12 different regions soils, the highest and lowest of Mg fertilizer use efficiency were WX (29.56) and YC (6.08) sites, respectively. Based on linear regression, the Mg fertilizer use efficiency in tea plant negatively correlated with soil initial total Mg content (Figure 3A, *r* = −0.63, *p* < 0.05), similarly, it negatively correlated with soil initial total N content (Figure 3B, *r* = −0.51, *p* < 0.05). However, there was no significant correlation between Mg fertilizer use efficiency and Ex-Mg in soils (*r* = −0.035, *p* = 0.91).

**Table 3:** Magnesium fertilizer use efficiency in typical tea plantation soils.

**Site**	**Mg fertilizer use efficiency in tea plant(%)**			
	**Root**	**Stem**	**Leaf**	**Total**
ChengDu (CD)	0.01	12.32	4.40	16.73
GuiLing (GL)	0.56	11.52	0.32	12.40
HuangGang (HG)	1.04	5.52	5.00	11.56
XinYang (XY)	0.01	11.48	0.24	11.73
XiShuangBanNa (XSBN)	1.84	10.60	4.16	16.60
WuXi (WX)	0.56	25.80	3.20	29.56
YiChang (YC)	2.16	1.00	2.92	6.08
ChangSha (CS)	0.88	4.80	5.00	10.68
NingDe (ND)	1.08	9.60	6.20	16.88
QingYuan (QY)	2.60	11.28	4.68	18.56
XiangXi (XX)	0.52	7.04	2.88	10.44
ChongQing (CQ)	4.28	11.12	7.12	22.52
Mean valve	1.30	10.17	3.84	15.31

Considering that quality traits of the crop are also vital for assessment of fertilizer use efficiency besides the growth and nutrient uptake, contents of chemicals related to tea quality have been analyzed. Compared with the control treatment, Mg input significantly increased the total contents of AA (by 7–88%, mean was 55%) but decreased those of TP (by 2–32%, mean was 15%). Therefore, the ratios of TP/AA in all samples were significantly decreased (by 24–60%, mean was 44%) with Mg fertilization (Supplementary Figure 3). Linear regression analysis showed that the ratio of TP/AA significantly and positively correlated (*r* = 0.66, *p* < 0.01, Supplementary Figure 4A) with soil initial total Mg contents value under Mg application, whereas, in the CK group, the ratio of TP/AA showed an insignificant correlation (*r* = −0.001, *p* = 0.99, Supplementary Figure 4A) with soil initial total Mg content. The correlation between TP/AA ratio and soil Ex-Mg were negligible (*r* = 0.09, 0.005; *p* = 0.06, 0.67 in CK and Mg group, respectively, Supplementary Figure 4B). On the other hand, without Mg application, the ratio of TP/AA in tea leaf negatively correlated with soil initial total N without Mg application (*r* = −0.73, *p* < 0.001, Supplementary Figure 4C). However, there is no significant correlation between TP/AA ratio and N contents in soil (*r* = 0.003, *p* = 0.75, Supplementary Figure 4C) with Mg application, indicating Mg fertilizer input in N-deficiency soils could have higher efficiency than in N-rich soils (Figures 3B,D).

## Discussion

### Plant-Available and Chemical-Available Mg in Tea Plantation Soil

It is generally accepted that soil chemical analysis is a common and rapid method for evaluating soil nutrient status. However, the efficiency of different extractions varied greatly due to their strengths of ion exchange (Iatrou et al., 2015; Zbíral, 2016). Based on comparing six different Mg extraction methods, Staugaitis and Rutkauskiene (2010) found that mild extraction procedures including CaCl_2_, KCl, NH_4_OAc, and Mehlich-3 method showed similar extraction effect. Nelson and Jones (1972) reported that the ammonium acetate (NH_4_Ac) method extracted more Mg from soils with ranging neutral to alkaline conditions, high clay, and high organic matter content than by double acid method, while the latter gives erroneously high results if soils contain dolomitic limestone or other acid-soluble Mg. In this study, four extraction methods including CaCl_2_, KCl, NH_4_OAc, and Mehlich-3 showed high correlations, and Ex-Mg was extracted most efficiently by Mehlich-3. Indicating the importance of choosing an appropriate extraction method for correct evaluation of the Mg availability to crops.

Soil chemical analysis only indicates the potential nutrient supply capacity of soil for the plant, and plant availability could be greatly affected by many biotic and abiotic factors, such as nutrient mobility, root growth, and rhizospheric microorganisms (Dragicevic et al., 2016; Yousaf et al., 2016; Soremi et al., 2017), which means a good extraction procedure should exhibit a high correlation with the total nutrient (Mg) uptake, while the absolute amount being extracted is irrelevant. However, a previous study suggested that there is often a poor relationship between the plant growth response and extractable nutrients in the soil (Ortas et al., 1999; Gransee and Führs, 2013). In this study, the concentrations of Mg in tea plant tissue were largely correlated with Ex-Mg extracted by CaCl_2_ than other chemical extractions (BaCl_2_, NH_4_OAC, and Mehlich-3), indicating that Ex-Mg extracted by CaCl_2_ strongly correlated with the plant availability of Mg in tea plantation soil. In agreement with Papenfuß and Schlichting (1979), results were that soil Ex-Mg extracted by CaCl_2_ can substantially contribute to plant Mg nutrition. Early results suggested that water-soluble and Ex-Mg fractions in soils could not always reflect the capacity of the crops to mine the soil (Gransee and Führs, 2013); therefore, evaluation of the contribution of different soil Mg pools to plant Mg nutrition is a great challenge. Although there have been no reports on the utilization rate of Mg nutrition in tea plants, different rates of Mg supply in the soil can be evaluated using selective extraction. Nevertheless, the correlation between chemical availability and plant response of Mg in this study is based on the variation of soil properties from 12 tea plantations, which makes it difficult to recommend extractable solutions for each specific soil type. Overall, individual extraction methods based on the relationship between chemical availability and plant response can produce optimal fertilizer recommendations. This implies that CaCl_2_ could be recommended for plant-available Mg extraction in tea plantation soil, further predicting the efficiency of Mg fertilizer application in tea plantations.

### Mg Fertilizer Use Efficiency and Contribution of the Soil to Plant Availability of Mg

Most nutrients in the soil cannot be directly absorbed and utilized by plants. Papenfuß and Schlichting (1979) suggested that the fixed Mg pool in soil can substantially contribute to plant Mg nutrition. However, inconsistent results on the contribution of the fixed soil Mg pool to plant Mg nutrition was also reported (Salmon and Arnold, 1963; Christenson and Doll, 1973; Kidson et al., 1975). It is worth noting that the ratio of polyphenols to amino acids highly correlated with the total Mg in the soil with the +Mg group (Supplementary Figure 4A), while it was uncorrelated with Ex-Mg (Supplementary Figure 4B). In addition, the Mg fertilizer also showed higher use efficiency in soil with low total Mg (Figure 3A). Concerning a poor relationship between the plant growth response and extractable nutrients in the soil (Ortas et al., 1999; Gransee and Führs, 2013), these results suggested that the desorption and mineralization of non-active Mg in soil may have potential effect on the plant availability of the Mg fertilizer. However, the mineralization of non-active Mg in soil always was affected by many other factors, such as soil texture. In general, compared with Ex-Mg, the total Mg showed a higher correlation to plant availability (including uptake and quality-improvement) of Mg application (Figures 3A,C). This may also be attributed to clay with Mg deficiency, which could absorb more Mg than clay with Mg adequacy. The latter will easily make more Mg available for plant uptake. However, the new knowledge should be confirmed by further studies on transformation in soil and metabolism in plants.

Fertilizers have higher plant availability because they are usually manufactured as a form that can be directly absorbed and utilized by the plant. However, plant availability can be affected by soil background nutrients. For example, plant availability of soil Mg and other cations is also related to the cation activity ratios in the soil solution. It was found that the approximate critical activity ratio for Mg/K (molar basis) should be 0.5 for good growth (Beckett, 1972). A positive effect of Mg on N uptake efficiency has been well-reviewed by Witold (2013). In a tea plantation, a previous study found that the yield and quality improvement effects of Mg application varied greatly in soils with different N levels (Ruan et al., 2012). In this study, compared to soils containing high N, soils with low N feature a higher plant availability of Mg fertilizers (Figure 3B). Moreover, there were significant and negative correlations between the ratio of polyphenol to amino acids and the total N contents of soil in the control group, while un-correlation was observed in the experimental group (Supplementary Figure 4C), suggesting that the application of Mg fertilizer may improve the utilization rate of N. Moreover, in this study, the application of Mg fertilizer had a great effect on decreasing the ratio of total polyphenol to amino acid in the tea leaves (Supplementary Figure 3), resulting in an increase in tea quality (Zhang and Ruan, 2016).

The content of Ex-Mg from different tea-producing areas varied greatly (30.71–245.04 mg kg^−1^). However, high consumption of Mg has been observed by comparing the status of Mg pre-tea cultivation and post-tea cultivation in soil, indicating that Mg depletion in tea soils should be a growing concern for tea production, although the potting system used in this experiment enlarged the nutrient cycle and Mg consumption. Moreover, acidic soil (pH ranged from 3.59 to 4.93) is not conducive to the absorption and utilization of Mg nutrition (Senbayram et al., 2016). In order to avoid situations of field scale Mg deficiency, precise knowledge of critical threshold values for Mg application in the tea plantation is required. In this study, the proportion of Mg taken upped in plant tissue from the Mg supply were 1.30, 10.17, and 3.84%, in root, stem, and leaf, respectively (Table 3). In case efficiency of Mg fertilizer use is the only consideration (the harvest part of 3.84%), about 7.8 kg Mg fertilizer is needed for 100 kg tea leaves (about 3.02 mg/g Mg) harvest (calculated as “100^*^3.02/3.84%”), 100 indicating “leaves harvest (100 kg),” 3.02 indicating “average value of Mg concentration in tea leaf after Mg application (3.02 g/kg ),” and 15.31 indicating “Mg fertilizer use efficiency in tea plant (15.31%).” However, the authors also found that Mg, which is almost four times (15.31/3.84) of the harvest amount, can be absorbed and utilized by plants and stored in the roots and stems. Considering reuse of storage nutrient is frequent in tea plants (Fan et al., 2020), our results show that extreme Mg deficiencies (based on both nutrient balance and tea quality) can be effectively avoided in tea plantation by application of about 2–3 kg (7.8/4) Mg year^−1^ (100 kg leaves harvest)^−1^.

## Conclusion

The plant availability of Mg fertilizer (6.08–29.56%) was quantified for the first time through a comparison of the uptake and quality-improvement effect in tea soils from 12 typical plantations in China. Moreover, the concentrations of exchangeable Mg, especially extracted by calcium chloride (CaCl_2_), highly correlated with the utilization of the tea plant. In addition, higher plant availability and quality-improvement effects (decreased the ratio of total polyphenol to amino acid) were observed in soils with a low level of total Mg content, as well as N deficiency status. Our results provide a potential theoretical guide of Mg fertilization in tea plantations.

## Data Availability Statement

The original contributions presented in the study are included in the article/Supplementary Material, further inquiries can be directed to the corresponding author/s.

## Author Contributions

QZ, DT, and XYi gathered samples. ML participated in the study design. QZ, SG, YH, YC, and KN performed data analysis. QZ and XYa interpreted the results and drafted the manuscript. JR and ML conceived of the study, provided funding, and gave guidance on experimental design. All authors read and approved the final manuscript.

## Conflict of Interest

The authors declare that the research was conducted in the absence of any commercial or financial relationships that could be construed as a potential conflict of interest.

## Publisher's Note

All claims expressed in this article are solely those of the authors and do not necessarily represent those of their affiliated organizations, or those of the publisher, the editors and the reviewers. Any product that may be evaluated in this article, or claim that may be made by its manufacturer, is not guaranteed or endorsed by the publisher.
