# Supplementary material for: Plant Availability of Magnesium in Typical Tea Plantation Soils
Source: Front Plant Sci. 2021 Aug 10;12:641501. doi: 10.3389/fpls.2021.641501 (PMC8383044; doi:10.3389/fpls.2021.641501)
Supplement: Supplementary file 1 [file Table_1.DOCX]

Table S1 information of soil samples from 12 tea plantations in China.

| **ID** | **Site** | **Province** | **Location** | **Soil type** | **P*** (mg/kg) | **K***(mg/kg) |
| --- | --- | --- | --- | --- | --- | --- |
| **CD** | ChengDu | Sichuan | 30.22 N, 103.60 E | Alfisols | 413.67±29.68 | 436.04±35.82 |
| **GL** | GuiLing | Guangxi | 25.06 N, 110.30 E | Oxisols | 184.96±2.8 | 301.54±8.09 |
| **HG** | HuangGang | Hubei | 30.50 N, 115.16 E | Alfisols | 182.58±2.67 | 298.38±4.13 |
| **XY** | XinYang | Henan | 32.34 N, 114.43 E | Alfisols | 40.98±2.09 | 333.96±3.87 |
| **XSBN** | XiShuangBanNa | Yunnan | 22.14 N, 100.82 E | Ultisols | 174.88±1.35 | 552.04±2.97 |
| **WX** | WuXi | Jiangshu | 31.36 N, 120.39 E | Alfisols | 994±3.81 | 712.92±4.73 |
| **YC** | YiChang | Hubei | 30.92 N, 111.05 E | Alfisols | 201.17±2.74 | 259.08±5.14 |
| **CS** | ChangSha | Hunan | 28.31 N, 112.69 E | Alfisols | 193.88±1.35 | 529.29±16.49 |
| **ND** | NingDe | Fujian | 26.69 N, 119.49 E | Oxisols | 681.71±9.64 | 110.89±7.36 |
| **QY** | QingYuan | Guangdong | 23.68 N, 113.05 E | Oxisols | 22.87±0.36 | 304.88±5 |
| **XX** | XiangXi | Hunan | 28.14 N, 109.73 E | Alfisols | 0.85±0.35 | 195.04±7.48 |
| **CQ** | ChongQing | Chongqing | 29.45 N, 106.73 E | Alfisols | 41.13±2.71 | 64.16±3.78 |

*Exchangeable phosphorus (P) and potassium (K) extracted using Mehlich-3 solutions.
