# Supplementary material for: Plant Availability of Magnesium in Typical Tea Plantation Soils
Source: Front Plant Sci. 2021 Aug 10;12:641501. doi: 10.3389/fpls.2021.641501 (PMC8383044; doi:10.3389/fpls.2021.641501)
Supplement: Supplementary file 2 [file Data_Sheet_1.docx]

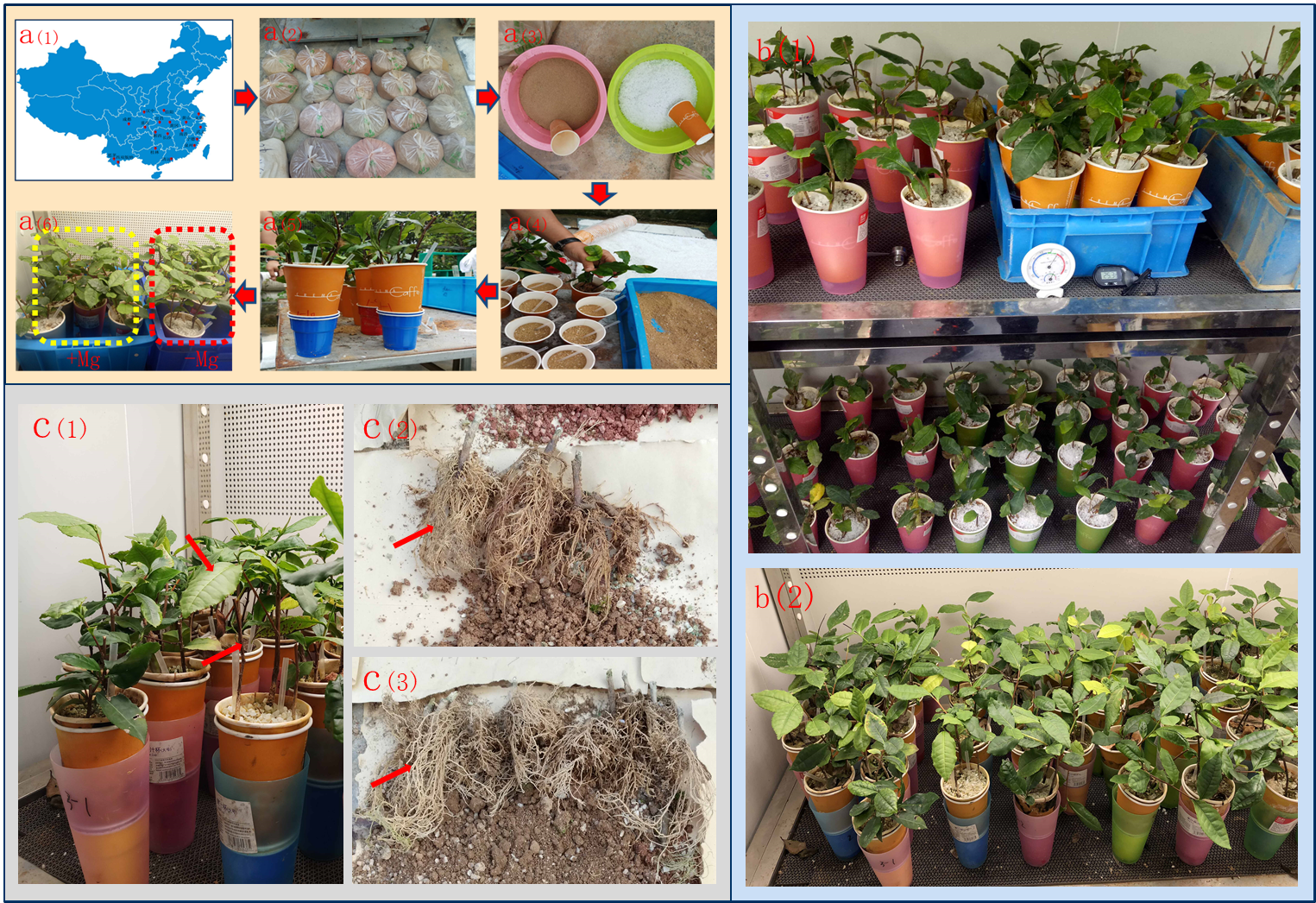


Figure S1. Experimental design(a1-a6), tea plant phenotypes (Longjing-43) grown in 12 typical tea plantation soils at 7 days (b1), 120 days (b2) and 180 days (c1-c3). Red arrow marked in c1, c2 and c3 shows the leaf, stem and root of tea plant, respectively.


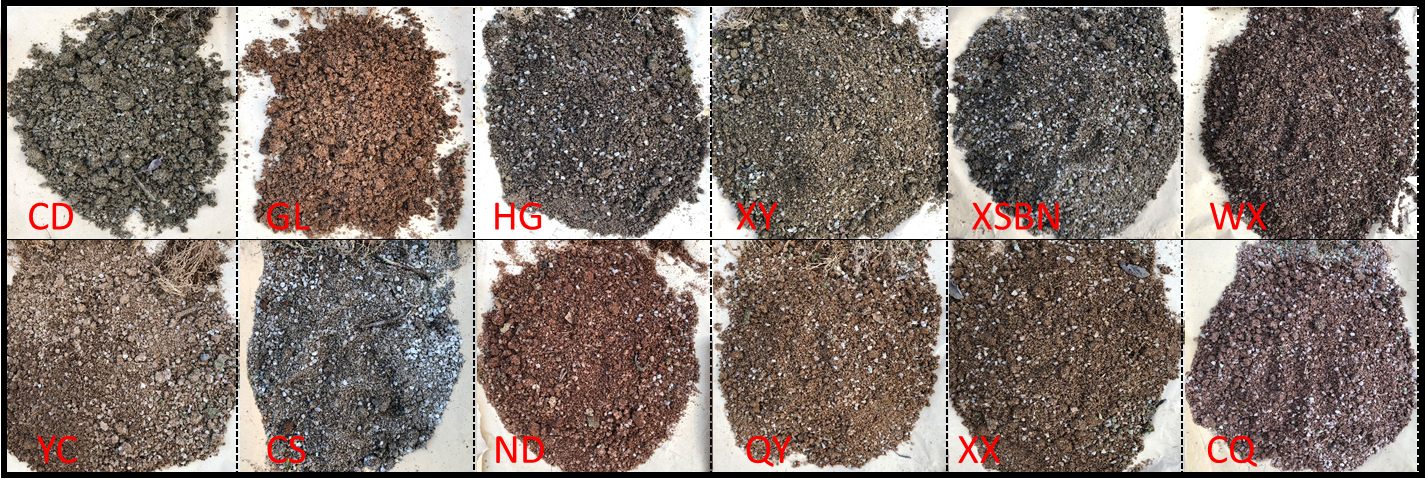


Figure S2. Appearance of Soil from 12 tea plantations in china after plant harvest. XY, Xsbn, WX, CD, HG, YC, QY, CS, GL, XX, ND, and CQ indicated that the soil has been collected in the site of Xinyang, Xishuangbanna, Wuxi, Chengdu, Huanggang, Qingyuan, Changsha, Guiling, Xiangxi, Ningde and Chongqing, respectively.


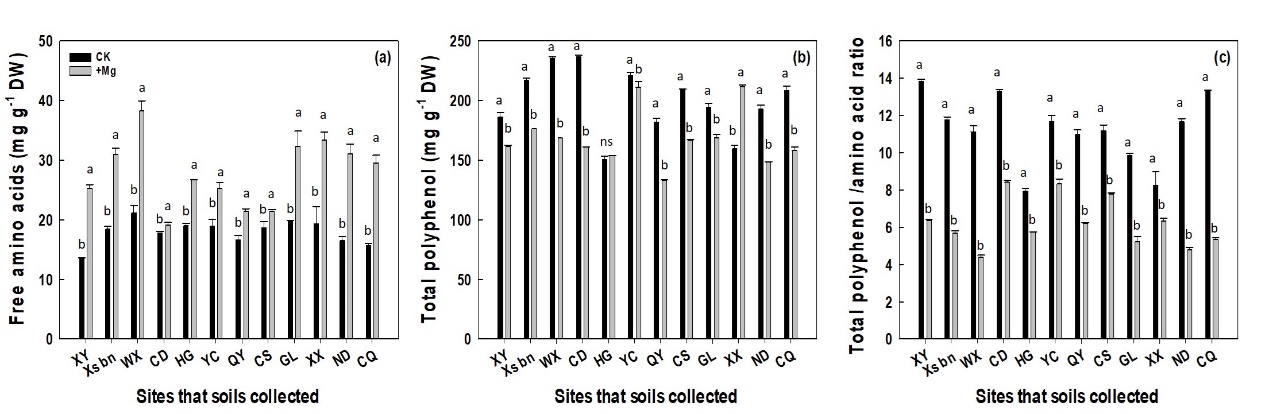


Figure S3. Effect of Mg supply on concentrations of free amino acid (a), polyphenol (b) and polyphenol/free amino acid (TP/AA) ratio (c) in tea leaf under Mg application (means ± SD, n=3). “ns” and different letters above the bar indicate insignificant and significant (p < 0.01) differences, respectively, between two magnesium treatments. XY, Xsbn, WX, CD, HG, YC, QY, CS, GL, XX, ND, and CQ indicated that the soil has been collected in the site of Xinyang, Xishuangbanna, Wuxi, Chengdu, Huanggang, Qingyuan, Changsha, Guiling, Xiangxi, Ningde and Chongqing, respectively.


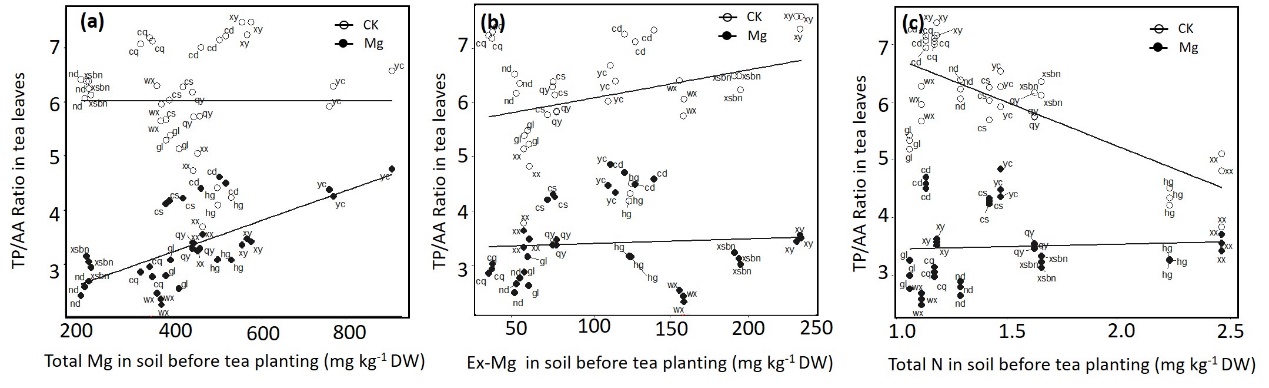


Figure S4. The correlation between polyphenol/free amino acid (TP/AA) ratio in tea leaves and content of total Mg (a), exchangeable Mg (b) or total nitrogen (N) in soil(c). Total Mg was extracted with nitric acid, perchloric acid and hydrofluoric acid from soil. XY, Xsbn, WX, CD, HG, YC, QY, CS, GL, XX, ND, and CQ indicated that the soil has been collected in the site of Xinyang, Xishuangbanna, Wuxi, Chengdu, Huanggang, Qingyuan, Changsha, Guiling, Xiangxi, Ningde and Chongqing, respectively.
